# A concise *in vitro* model for evaluating interactions between macrophage and skeletal muscle cells during muscle regeneration

**DOI:** 10.3389/fcell.2023.1022081

**Published:** 2023-05-18

**Authors:** Naoya Kase, Yohko Kitagawa, Akihiro Ikenaka, Akira Niwa, Megumu K. Saito

**Affiliations:** Department of Clinical Application, Center for IPS Cell Research and Application, Kyoto University, Kyoto, Japan

**Keywords:** skeletal muscle regeneration, innate immune memory, macrophages, regeneration efficiency, *in vitro* disease modeling

## Abstract

Skeletal muscle has a highly regenerative capacity, but the detailed process is not fully understood. Several *in vitro* skeletal muscle regeneration models have been developed to elucidate this, all of which rely on specialized culture conditions that limit the accessibility and their application to many general experiments. Here, we established a concise *in vitro* skeletal muscle regeneration model using mouse primary cells. This model allows evaluation of skeletal muscle regeneration in two-dimensional culture system similar to a typical cell culture, showing a macrophage-dependent regenerative capacity, which is an important process in skeletal muscle regeneration. Based on the concept that this model could assess the contribution of macrophages of various phenotypes to skeletal muscle regeneration, we evaluated the effect of endotoxin pre-stimulation for inducing various changes in gene expression on macrophages and found that the contribution to skeletal muscle regeneration was significantly reduced. The gene expression patterns differed from those of naive macrophages, especially immediately after skeletal muscle injury, suggesting that the difference in responsiveness contributed to the difference in regenerative efficiency. Our findings provide a concise *in vitro* model that enables the evaluation of the contribution of individual cell types, such as macrophages and muscle stem cells, on skeletal muscle regeneration.

## 1 Introduction

Skeletal muscle is a highly regenerative tissue (Tedesco et al., 2010). PAX7^+^ satellite cells represent the stem cells responsible for skeletal muscle regeneration and they reside in the tissue (Mauro, 1961; Seale et al., 2000). When the skeletal muscle is injured, activated satellite cells proliferate and compensate for the damaged skeletal muscle by fusing together (Almada and Wagers, 2016). This process results in efficient regeneration because of the potential of satellite cells and their interaction with various cells within the skeletal muscle niche, such as fibro-adipogenic progenitors (Joe et al., 2010; Uezumi et al., 2010), regulatory T-cells (Burzyn et al., 2013; Castiglioni et al., 2015), and macrophages (Segawa et al., 2008; Saclier et al., 2013). In particular, macrophages are known to strongly support skeletal muscle regeneration because of their ability to phagocytose injured tissues (McLennan, 1996) and release specific humoral factors (Cantini et al., 1995; Shen et al., 2008; Lu et al., 2011; Baht et al., 2020). However, owing to the complexity of the skeletal muscle niche and the limited number of models that can adequately demonstrate the contribution of macrophages to skeletal muscle regeneration, further studies are necessary.

Skeletal muscle regeneration has been studied mainly via *in vivo* experiments because of the importance of cell–cell interactions and spatial organization within the niche. Specifically, skeletal muscle injury is induced by treatment with cardiotoxin or barium chloride (BaCl_2_), which have myotoxic effects, and subsequent regeneration is observed (Hardy et al., 2016). However, the use of animal models is affected by the low throughput and complexity of genetic manipulation. Therefore, several *in vitro* skeletal muscle regeneration models have been established in recent years that use three-dimensional culture methods (Juhas et al., 2014; Juhas et al., 2018; Fleming et al., 2019; Fleming et al., 2020; Tiburcy et al., 2019) or two-dimensional (2D) culture with specialized scaffolds (Davoudi et al., 2022) to accurately mimic the regeneration observed *in vivo*. However, the inaccessibility of specialized culture methods and techniques limits their application to general experiments currently used in many skeletal muscle studies *in vitro* and prevents the widespread use of these models. Therefore, there is a need for a skeletal muscle regeneration model that can be established using equipment and techniques such as those used in general cell culture.

Macrophages are immune cells that play a central role in the innate immune system and tissue repair (Wynn and Vannella, 2016). Based on their various roles, their phenotypic plasticity, represented by M1–M2 macrophages (Sica and Mantovani, 2012), has been widely studied. The concept of “innate immune memory” has been recognized, in which macrophages are subjected to epigenetic imprinting by specific antigens such as endotoxins and their immune responses are stored (Foster et al., 2007; Quintin et al., 2012; Saeed et al., 2014). Thus, macrophage plasticity and innate immune memory further complicate the discussion on their phenotypes. Recent studies have shown that muscle damage in Duchenne muscular dystrophy results in innate immune memory (Bhattarai et al., 2022). This suggests a crosstalk between innate immune memory and tissue injury/regeneration. Nevertheless, contrary to the aforementioned study, it has not been investigated how macrophages, in which innate immune memory is formed, are phenotypically altered in terms of tissue repair.

Here, we present an *in vitro* skeletal muscle regeneration model developed using mouse primary cells without specialized culture methods and techniques. The model allows for macrophage depletion and addition via cell sorting, allowing the evaluation of how macrophages in various phenotypes contribute to skeletal muscle regeneration. Specifically, macrophages stimulated by lipopolysaccharides (LPS) were found to contribute less to skeletal muscle regeneration. Our findings provide a simple method to study *in vitro* skeletal muscle regeneration and suggest an impact of innate immune memory on tissue repair.

## 2 Material and methods

### 2.1 Experimental animals

C57BL/6 mice were purchased from Japan SLC (Shizuoka, Japan). P6–8 C57BL/6 mice were used for collecting skeletal muscle samples and mice aged 8–12 weeks for collecting bone marrow cells and injured skeletal muscle. Animal experiments were performed according to protocols approved by the Animal Research Committee of Kyoto University.

### 2.2 *In vitro* skeletal muscle differentiation

Skeletal muscle was collected from the entire lower extremities of the mice. The collected skeletal muscle was homogenized using scissors in Dulbecco’s modified Eagle’s medium (DMEM) (08459-64; Nacalai Tesque, Kyoto, Japan) containing 20% fetal bovine serum (FBS) (10437-028; Gibco, Waltham, MA, United States), 1/100 dilution of antibiotic-antimycotic (15240; Gibco), and 5 mg/mL collagenase (09353-04; Nacalai Tesque), followed by incubation at 37°C for 1 h. Homogenized samples were washed with phosphate-buffered saline (PBS) (14249-24; Nacalai Tesque), distributed equally among four 10-cm culture dishes, and cultured in DMEM containing 20% FBS (expansion medium) for expansion. Culture dishes (10 cm) were coated overnight with a 1/50 dilution of Matrigel^®^ Growth Factor Reduced (354230; Corning, Corning, NY, United States) prior to sample distribution. The medium was changed after 2 days. After 3 days of culture, the cells were reseeded into Matrigel-coated well plates at a concentration of 2 × 10^5^ cells/cm^2^ via detachment using 0.25% Trypsin-EDTA (25200-072; Gibco). The medium was changed to DMEM containing 2% horse serum (H1270; Sigma–Aldrich, St. Louis, MO, United States) (differentiation medium: DM) to initiate differentiation on the next day. The medium was changed every 2 days, and differentiated skeletal muscle was obtained 6 days after the initiation of differentiation (0 days post-injury: 0 dpi).

### 2.3 Differentiation of bone marrow-derived macrophages (BMDMs)

Bone marrow cells were collected by flushing the femurs and tibias using PBS. After washing the collected cells in PBS, cells were differentiated in DMEM containing 10% FBS, 1/100 dilution of antibiotic-antimycotic, and 40 ng/mL macrophage colony-stimulating factor (416-ML; R&D Systems, Minneapolis, MN, United States), and the medium was changed every 3 days. After 5–7 days from the start of differentiation, the attached BMDMs were collected using Accumax™ (17087-54; Nacalai Tesque). To induce innate immune memory, BMDMs were treated with 50 ng/mL LPS (tlrl-peklps; InvivoGen, San Diego, CA, United States) for 24 h before use.

### 2.4 Macrophage depletion

Macrophages were depleted using magnetic cell sorting before the expanded cells were reseeded. Expanded samples were labeled with CD45 microbeads (130-052-301; Miltenyi Biotec, Bergisch Gladbach, Germany) at 4°C for 1 h after washing in PBS. Labeled samples were sorted using an autoMACS^®^ Pro Separator (Miltenyi Biotec) and negative fractions were collected.

### 2.5 BaCl_2_ injury and regeneration

To induce skeletal muscle injury, distilled water (10977; Invitrogen, Waltham, MA, United States) containing 12% BaCl_2_ (03811; Nacalai Tesque) was added to the medium at 0 dpi at a final concentration of 0.48%. After 6 h of incubation at 37°C (0.25 dpi), the medium was aspirated, washed in PBS, and replaced with DM for regeneration. The medium was changed every 2 days and regeneration was induced at 6 dpi.

### 2.6 Immunocytochemistry and measurements

Samples were fixed with 4% paraformaldehyde (091554; Nacalai Tesque) for 30 min and methanol (21915; Nacalai Tesque) for 10 min at room temperature (22°C–25°C) and blocked with 1xBlock Ace (UK-B80; DS Pharma Biomedical, Osaka, Japan) before overnight incubation with α-actinin antibodies (ab9465; Abcam, Cambridge, United Kingdom) diluted in PBS containing 0.3% Triton^®^ X-100 (A16046; Alfa Aesar, Ward Hill, MA, United States) and 1% bovine serum albumin (A4503; Sigma–Aldrich) (dilution buffer) at 4°C. The samples were washed in PBS and incubated with Alexa Fluor^®^ 594-conjugated anti-mouse IgG (8890; Cell Signaling Technology, Danvers, MA, United States) diluted in dilution buffer for 1 h at room temperature. Next, 4′,6-diamidino-2-phenylindole dihydrochloride (DAPI) (32670; Sigma–Aldrich) was used to counterstain the nuclei. The samples were imaged using an FV3000 microscope (Olympus, Tokyo, Japan). Transmitted light images and their merged with immunofluorescence were taken by BZ-X800 (Keyence, Osaka, Japan). The images were analyzed using ImageJ software (National Institutes of Health, Bethesda, MD, United States). All α-actinin observation images were binarized and their positive areas were quantified. Positive areas containing three or more nuclei were then determined by handling and quantified as myotube areas. Debris areas were defined as α-actinin-positive areas minus the myotube areas. Quantitative value was determined by averaging the eight images obtained from randomly selected fields per one sample.

### 2.7 RNA isolation and quantitative polymerase chain reaction (qPCR)

Total RNA was column-purified using an RNeasy Mini Kit (74106; Qiagen, Hilden, Germany) and treated with RNase-free DNase (79254; Qiagen). Purified RNA was reverse-transcribed using PrimeScript RT Master Mix (RR037A; Takara Bio, Kusatsu, Japan) according to the manufacturer’s protocol. Quantitative polymerase chain reaction was performed using StepOnePlus (Applied Biosystems, Waltham, MA, United States) and TB Green Premix Ex Taq II (RR820A; Takara Bio). Primer sequences used in this study are listed in Table 1.

**TABLE 1:** The list of primers for the qPCR.

Gene	Forward	Reverse
*Ccl2*	5' CAGGTCCCTGTCATGCTTCT	5' GTGGGGCGTTAACTGCATCT
*Il6*	5' CCGGAGAGGAGACTTCACAG	5' CAGAATTGCCATTGCACAAC
*Metrnl*	5' GTGGGCTCAGTCGCTCTATC	5' CAATGGGTCAGGGCATCGTT
*Col6a1*	5' GATCCCGCCCTTGGTTTGTA	5’ AGGAGGAAGACGAGATGGCT
*Tnc*	5’ GAGACCTGACACGGAGTATGAG	5’ CTCCAAGGTGATGCTGTTGTCTG
*Gapdh*	5' ATGACATCAAGAAGGTGGTG	5' CATACCAGGAAATGAGCTTG

### 2.8 Flow cytometry

Flow cytometry was performed using FACS Aria™ II (BD Biosciences, Franklin Lakes, NJ, United States). Samples were detached using 0.25% trypsin-EDTA and single cells were collected by filtration using a pluriStrainer Mini 40 µm (43-10040; pluriSelect, Leipzig, Germany). Cells were counted using a Countess^®^ II FL automated cell counter (Thermo Fisher Scientific, Waltham, MA, United States), following staining with Trypan blue. Single cells were stained in PBS containing 2% FBS, 2 mM EDTA (15575; Gibco), and TruStain FcX™ Antibody (101320; BioLegend, San Diego, CA, United States) for 30 min on ice using antibodies, followed by DAPI staining for dead cell detection. The following antibodies were used: PE anti-mouse Ly-6G antibody (127608; BioLegend), fluorescein isothiocyanate-conjugated anti-mouse CD45 antibody (103108; BioLegend), APC anti-mouse/human CD11b antibody (101212; BioLegend), PE rat IgG2a, κ isotype control antibody (400508; BioLegend), fluorescein isothiocyanate-conjugated rat IgG2b, κ isotype control antibody (400606; BioLegend), and APC rat IgG2b, κ isotype control antibody (400612; BioLegend). Data analyses were performed using FlowJo software (BD Biosciences).

### 2.9 RNA sequencing

Fluorescence-activated cell sorting-sorted cells were lysed in RLT buffer (79216; Qiagen) and RNA was extracted using RNAClean XP beads (A66514; Beckman Coulter, Brea, CA, United States). Reverse transcription was performed using the SMART-Seq v4 Ultra Low Input RNA Kit (Takara Bio). cDNA was then fragmented using a Covaris Focused-ultrasonicator M220 (M&S Instruments Inc., Osaka, Japan). The library was constructed using a SMARTer ThruPLEX DNA-seq 48S Kit (Takara Bio) and sequenced on a NextSeq 500 System (Illumina, San Diego, CA, United States) with 75-bp single-end reads. No technical replicates were performed. The reads were trimmed using Cutadapt (version 1.15) and mapped to the mouse genome mm10 using Hisat2 (version 2.1.0). Tags were counted using featureCounts (version 1.6.0) (Liao et al., 2014) and normalized using EdgeR for principal component analysis or DEseq2 for differentially expressed gene (DEG) analyses within iDEP.951 (Ge et al., 2018). Genes with a false discovery rate <0.1 and fold change >2 were considered DEGs. For functional analysis, the DEGs were subjected to gene ontology (GO) biological process enrichment. Visualization of each analysis was conducted within iDEP.951 or shynyGO 0.76 (Ge et al., 2020). RNA-Seq data sets have been deposited in National Center for Biotechnology Information Sequence Read Archive under accession number PRJNA880816. Gene set enrichment analysis (GSEA) (Subramanian et al., 2007) was performed using GSEA software 4.3.6 (Broad Institute, Cambridge, MA, United States). We used built-in M5 curated gene sets (Molecular Signatures Database 3.0: www.broadinstitute.org/gsea/msigdb). The statistical significance of GSEA was analyzed using 1000 permutations following default setting. Enrichment was compared among Naive and LPS macrophages at 1 dpi in this study.

### 2.10 *In vivo* skeletal muscle injury and transwell assay

*In vivo* skeletal muscle injury and its exposure to macrophages was carried out according to a previous report with minor modifications (Paliwal et al., 2012)*. In vivo* skeletal muscle injury was induced through an injection of 1.2% BaCl_2_ into the right side of tibialis anterior (TA) muscle using a 29-gauge needle. Injured TA muscles collected the next day were carefully homogenized by scissors in 1 mL of DMEM +20% FCS. The homogenized tissue was immediately seeded directly onto a 0.4 μm pore size Transwell membrane (353090, Corning) and exposed indirectly to the underlying BMDMs. Exposed BMDMs were collected after 24 h and used for co-culture with skeletal muscle *in vitro*.

### 2.11 Enzyme-linked immunosorbent assay (ELISA)

After 5 days of differentiation of BMDMs, they were treated with or without 50 ng/mL LPS for 24 h as first stimulation, and after washing and another 6 days of culture, cells were reseeded at 1 × 10^4^ cells/well in 96-well plates. Reseeded cells were stimulated with 50 ng/mL LPS for 24 h as a secondary stimulus or unstimulated, and the supernatant was collected and assayed. The concentrations of proinflammatory cytokines were measured by using a LEGENDplex Mouse Anti-Virus Response Panel (740621, BioLegend) according to the manufacturer’s protocol. Cytokine concentrations were quantified using Guava easyCyte (Luminex, Austin, TX, United States). After seeding 1 × 10^4^ BMDMs into each well of 96-well plates, LPS at 50 ng/mL were added as the second stimulation. After culturing for 24 h, the supernatants were sampled and assayed.

### 2.12 Statistical analyses

All statistical analyses were performed using GraphPad Prism (GraphPad Software, San Diego, CA, United States). *p* < 0.05 was considered statistically significant. Data with two groups were analyzed by Student’s *t*-tests and three or more groups by one way-analysis of variance (ANOVA) followed by Šidák *post hoc* tests.

## 3 Results

### 3.1 *In vitro* differentiated skeletal muscle derived from mouse primary cells enabled regeneration following BaCl_2_ injury

**FIGURE 1:**
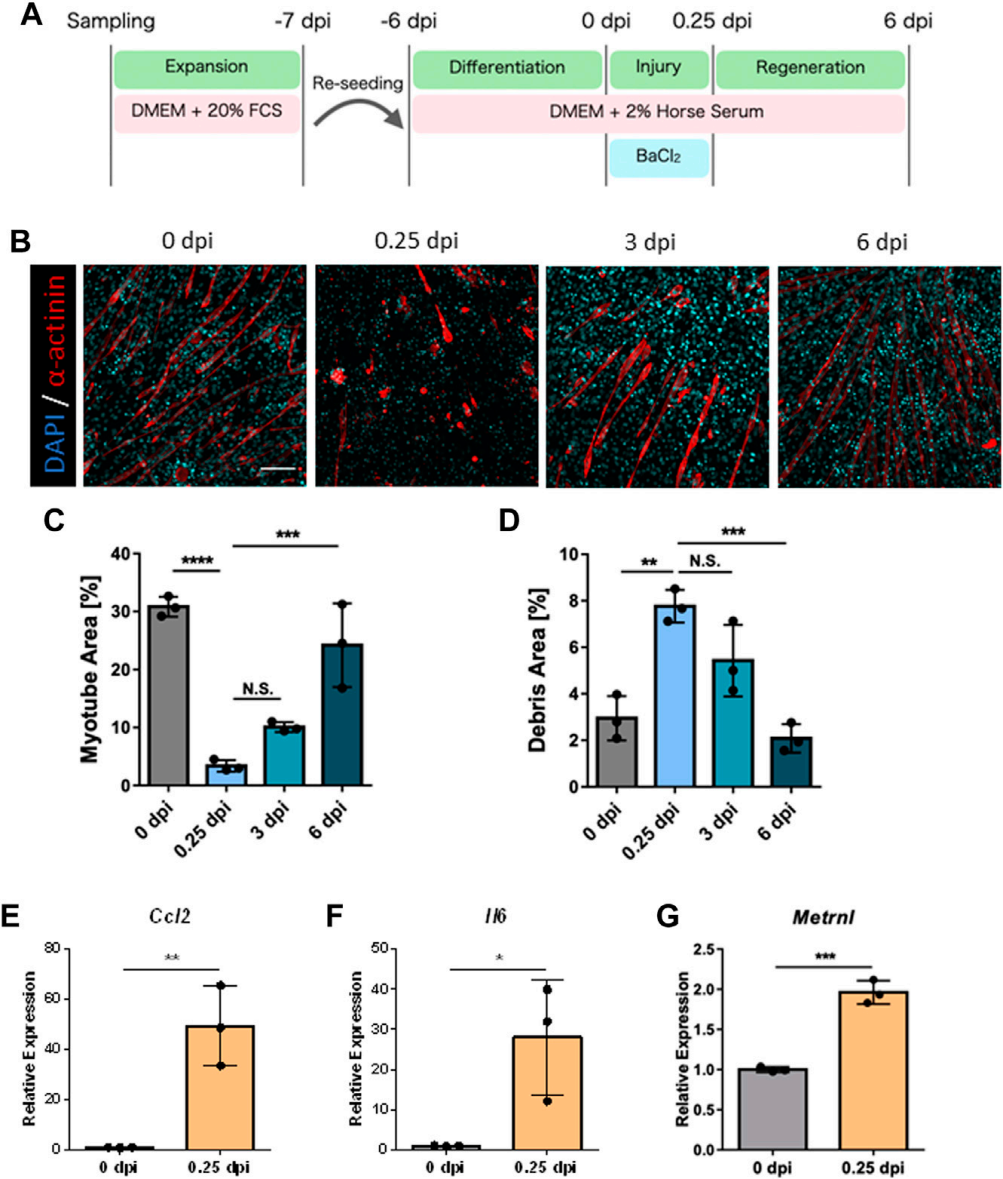
Observation of skeletal muscle regeneration *in vitro*. **(A)** Schematic representation of the time course and culture conditions. **(B)** Time-dependent representative images of myotube formation. Scale bar indicates 100 μm. **(C, D)** Quantification of **(C)** myotube and **(D)** debris area (*N* = 3). Statistical analysis was performed using one-way ANOVA followed by Šidák *post hoc* tests: **p < 0.01, ***p < 0.005, ****p < 0.001. **(E–G)**: Relative expression levels of **(E)** *Ccl2*, **(F)** *Il6*, and **(G)** *Metrnl* (*N* = 3). Expression levels were normalized to glyceraldehyde 3-phosphate dehydrogenase gene expression. Statistical analysis was performed using the Student’s *t*-test: *p < 0.05, **p < 0.01, ***p < 0.005. Data are presented as the mean ± standard deviation of biologically independent samples from different mice. DMEM, Dulbecco’s modified Eagle medium; DAPI, 4′,6-diamidino-2-phenylindole; *Ccl2*, chemokine (C-C motif) ligand 2; *Il6,* interleukin 6; *Metrnl,* meteorin-like.

First, to establish a simple method for skeletal muscle regeneration, we examined whether differentiated skeletal muscles derived from mouse primary cells possess regenerative potential. Since the microenvironment consisting of surrounding cells contributes to skeletal muscle regeneration efficiency, we collected all cells present in the neonatal skeletal muscle by digesting only the tissue. Hence, the harvested cell population contains immune cells, fibroblasts, and endothelial cells in addition to myoblasts. Collected cells were expanded in the expansion medium and cultured in DM for 6 days to obtain *in vitro* differentiated skeletal muscle (Figures 1A, B). Treatment of differentiated skeletal muscle with BaCl_2_ resulted in disruption of skeletal muscle structure, decrease in myotube area, and an increase in amount of debris based on α-actinin staining (Figures 1A–D; Supplementary Figure S1). After washing out BaCl_2_ and culturing in DM for 6 days, regeneration of skeletal muscle structures was observed (Figures 1A, B; Supplementary Figure S1). At 6 dpi, the myotube area significantly recovered to an equivalent level to that at 0 dpi, and the debris area also decreased accordingly (Figures 1C, D). These results indicate that *in vitro* differentiated skeletal muscle derived from mouse primary cells is capable of regeneration, suggesting that regeneration by this method provides a simple model for the evaluation of regenerative efficacy.

### 3.2 Myokines specific to skeletal muscle injury were expressed *in vitro*

Skeletal muscle releases specific myokines upon injury, which contribute to regeneration. However, skeletal muscle injury induced by BaCl_2_ treatment in 2D cultures of the myoblast cell line C_2_C_12_ does not fully reproduce the expression of these myokines (Fleming et al., 2019). Therefore, we examined whether our model, using primary mouse cells, shows increased myokine expression after injury. Gene expression levels were quantified for chemokine (C-C motif) ligand 2, interleukin 6, and meteorin-like, representative myokines associated with skeletal muscle injury (Fleming et al., 2019; Baht et al., 2020) by qPCR, and a significant increase in expression was observed at 0.25 dpi for all myokines (Figures 1E–G). These results indicated that our model reflects the response to injury in terms of myokine expression.

### 3.3 Macrophages contributed to regenerative efficacy *in vitro*

**FIGURE 2:**
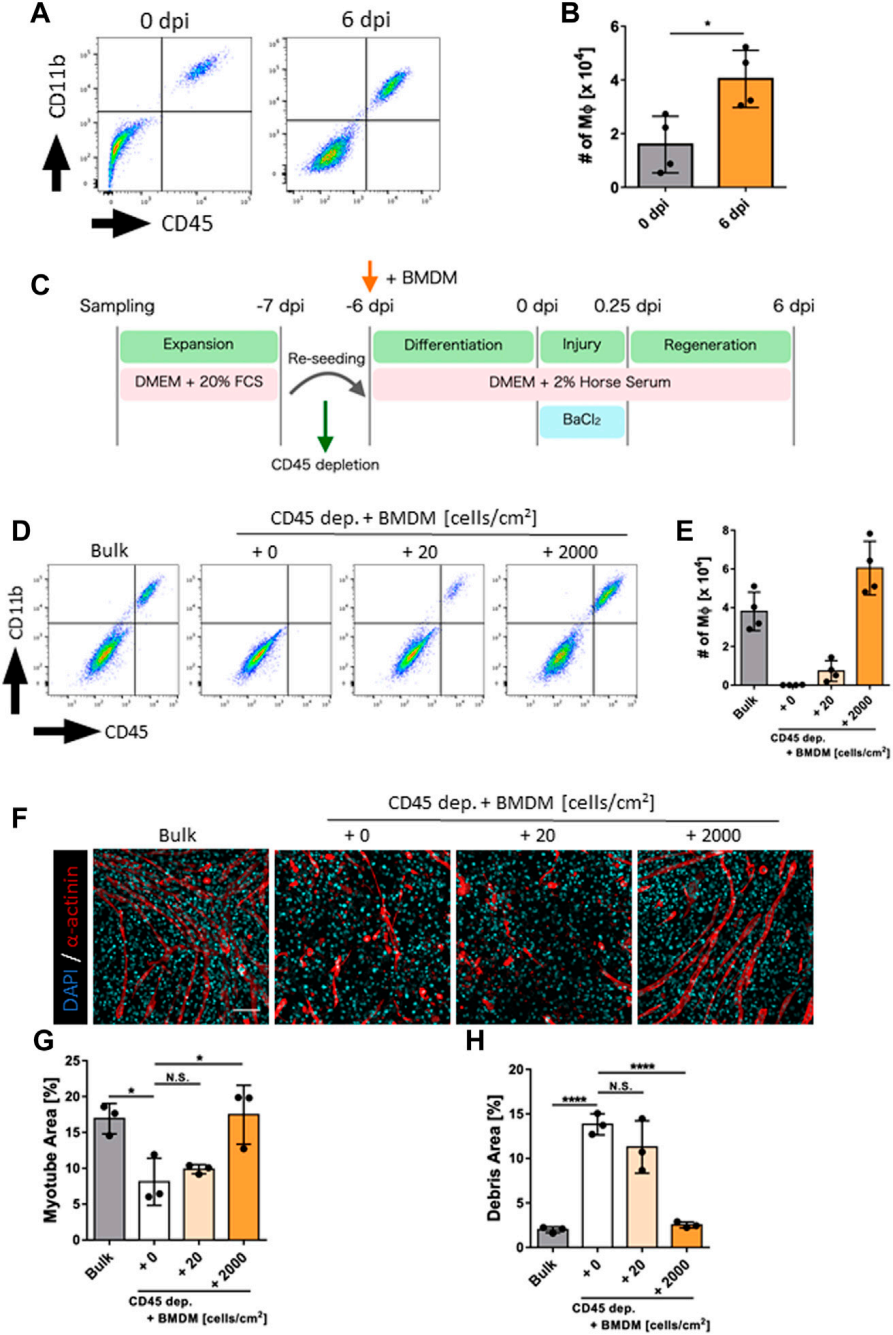
Evaluation of macrophage contribution to skeletal muscle regeneration *in vitro*. **(A)** Flow cytometry analysis for the detection of macrophages at 0 and 6 dpi. **(B)** The number of macrophages at 0 and 6 dpi (*N* = 4). Statistical analysis was performed using the Student’s *t*-test: *p < 0.05. **(C)** Schematic representation of the time course and culture conditions. **(D)** Flow cytometry analysis to detect macrophages after depletion and addition of macrophages. **(E)** Number of macrophages at 6 dpi (*N* = 4). **(F)** Representative images of myotube formation after the depletion and addition of macrophages at 6 dpi. Scale bar indicates 100 μm. **(G, H)** Quantification of **(G)** myotube and **(H)** debris areas (*N* = 3). Statistical analysis was performed using one-way ANOVA followed by Šidák *post hoc* tests: *p < 0.05, ****p < 0.001. Data are presented as the mean ± standard deviation of biologically independent samples from different mice. BMDMs, bone marrow-derived macrophages; DMEM, Dulbecco’s modified Eagle medium; DAPI, 4′,6-diamidino-2-phenylindole; dpi, days post-injury.

Since macrophages are major contributors to skeletal muscle regeneration, we examined the presence of macrophages in our model. Flow cytometry analysis detected Ly6G^−^CD45^+^CD11b^+^ macrophages at 0 and 6 dpi (Figure 2A; Supplementary Figure S2). Furthermore, the number of macrophages increased during the regeneration process (Figure 2B). The detected macrophages were speculated to be tissue resident in the skeletal muscle of neonatal mice, and their self-renewal capacity may therefore increase during regeneration. Next, we depleted macrophages from pre-seeded cells via magnetic cell sorting and added BMDMs to the depleted samples to determine whether existing macrophages contribute to regeneration (Figure 2C). The efficiency of macrophage depletion and BMDM engraftment at 6 dpi via flow cytometry analysis showed almost complete depletion of macrophages and engraftment of added BMDMs (Figures 2D, E). Quantification of myotube area under this condition at 6 dpi revealed that the regeneration efficiency was significantly decreased by the depletion of macrophages and restored by the addition of BMDMs (Figures 2F, G; Supplementary Figure S3). Correspondingly, the debris area was significantly increased by the depletion of macrophages and restored by the addition of BMDMs (Figure 2H). Therefore, macrophages contributed to the efficiency of skeletal muscle regeneration in our model. Furthermore, our model can be used to evaluate functional differences to determine their contribution to skeletal muscle regeneration.

### 3.4 LPS pre-stimulation decreased the contribution of macrophages to skeletal muscle regeneration

Based on the above results, we hypothesized that this model could be used to evaluate the differential function of macrophages during skeletal muscle regeneration. To proof this concept, we next investigated whether the LPS-stimulated macrophages influence their contribution to skeletal muscle regeneration. Although LPS stimulation induces the activation of macrophages, it also causes global epigenetic changes, resulting in differential responsiveness to subsequent secondary stimulation known as innate immune memory (O’Carroll et al., 2014; Zubair et al., 2021). However, the effect of LPS stimulation on the efficiency of tissue repair remains unknown. Therefore, we determined the effect of LPS-induced macrophage activation (Seeley and Ghosh, 2017) on the efficiency of skeletal muscle regeneration using our model. Macrophages exposed to endotoxins and inflammatory cytokines are known to acquire an innate immune tolerance in which their secondary reactivity is decreased (Foster et al., 2007; Quintin et al., 2012; Saeed et al., 2014), but this is known to be culture condition-dependent and transient (O’Carroll et al., 2014). Assuming the system in which LPS is stimulated into BMDMs just prior to starting co-culture with skeletal muscle, we first evaluated the activation state of BMDMs at 6 days after LPS transient stimulation since BMDMs are exposed to muscle injury after 6 days co-culture in our model. BMDMs transiently stimulated with LPS 6 days prior to secondary LPS stimulation secreted proinflammatory cytokines more prominently than those with pre-stimulation (Supplementary Figures S4A, B), indicating that macrophages have acquired innate immune memory without tolerance over the time course of this assay.

**FIGURE 3:**
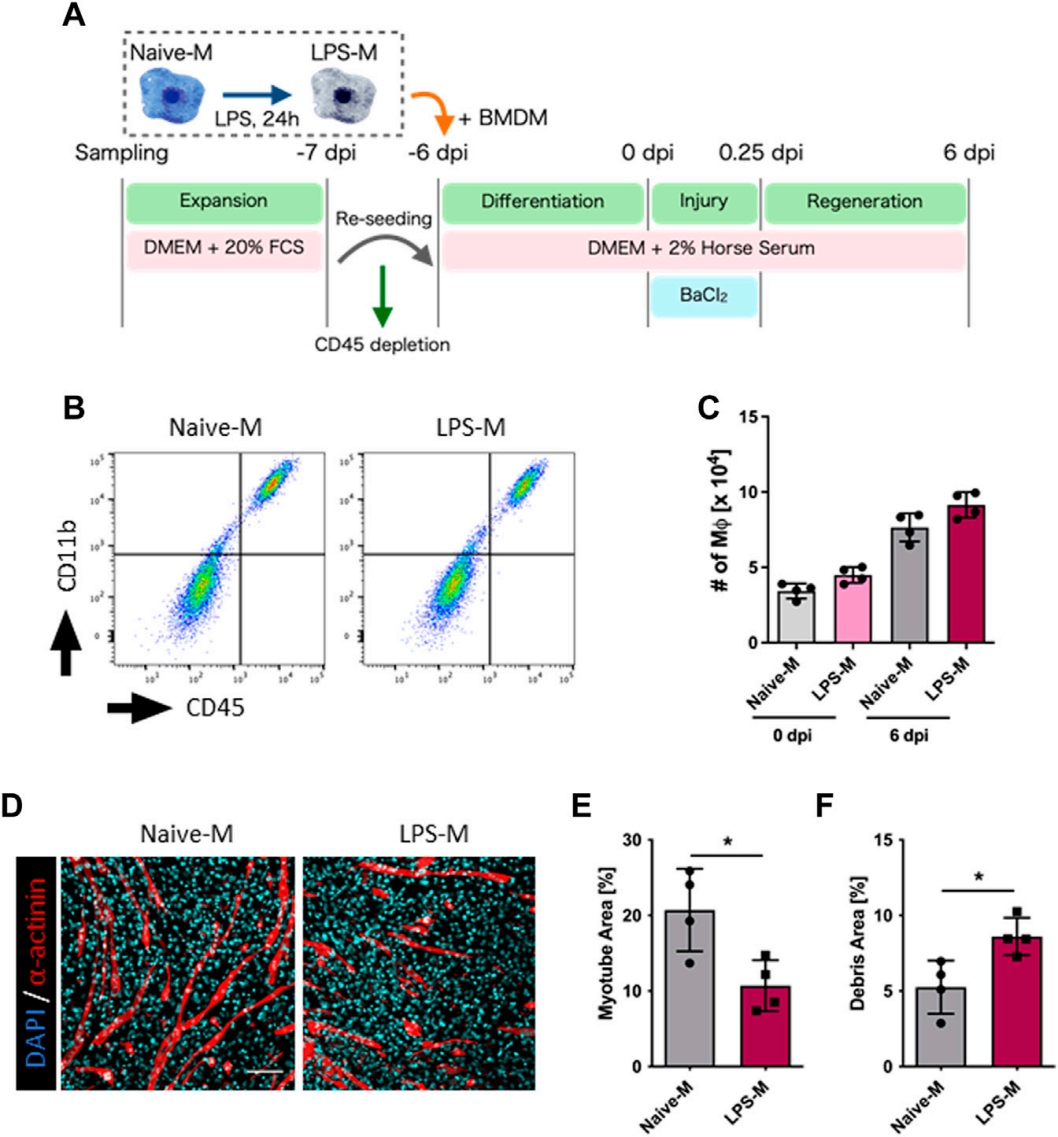
Evaluation of regeneration efficiency in LPS-pre-stimulated macrophages. **(A)** Schematic representation of the time course and culture conditions. **(B)** Flow cytometry analysis for the detection of Naive-M and LPS-M at 6 dpi. **(C)** The number of Naive-M and LPS-M at 0 and 6 dpi (*N* = 4). **(D)** Representative images of myotube formation after addition of Naive-M and LPS-M at 6 dpi. Scale bar indicates 100 μm. **(E, F)** Quantification of **(E)** myotube and **(F)** debris areas (*N* = 4). Statistical analysis was performed using the Student’s *t*-test: *p < 0.05. Data are presented as the mean ± standard deviation of biologically independent samples from different mice. DMEM, Dulbecco’s modified Eagle medium; DAPI, 4′,6-diamidino-2-phenylindole; dpi, days post-injury.

To evaluate the effect of innate immune memory granted by LPS stimulation, unstimulated BMDMs (Naive-M) and LPS-pre-stimulated BMDMs (LPS-M) were added to the culture environment after depletion of endogenous macrophages and evaluated for skeletal muscle regeneration (Figure 3A). Their presence in the culture environment was analyzed via flow cytometry and both were found to be engrafted before and after regeneration (Figures 3B, C). The myotube area at 6 dpi was quantified under these conditions and was significantly reduced in LPS-M (Figures 3D, E; Supplementary Figure S5). Correspondingly, the debris area increased in LPS-M (Figures 3D, F; Supplementary Figure S5).

Although stimulation of macrophages with LPS is a typical model mimicking infectious and inflammatory conditions, it is meaningful to evaluate the contribution of macrophages to skeletal muscle regeneration when repeatedly exposed to muscle-derived damage-associated molecular patterns (DAMPs) as more physiologic conditions that may occur. To expose macrophages to muscle-derived DAMPs, we collected *in vivo* BaCl_2_-injured skeletal muscle and co-cultured it with macrophages while preventing cell-to-cell migration using Transwell. The exposed macrophages were then co-cultured with skeletal muscle to evaluate their effects on regeneration efficiency. However, no difference was observed in the contribution to skeletal muscle regeneration efficiency between naive macrophages and those exposed to injured muscle in our model (Supplementary Figures S6A, B). Therefore, under the current experimental conditions, there seems to be a specific role for LPS stimulation to impart macrophages the ability to regenerate muscle.

#### 3.5 LPS pre-stimulation altered the gene expression pattern of macrophages responding to skeletal muscle injury

**FIGURE 4:**
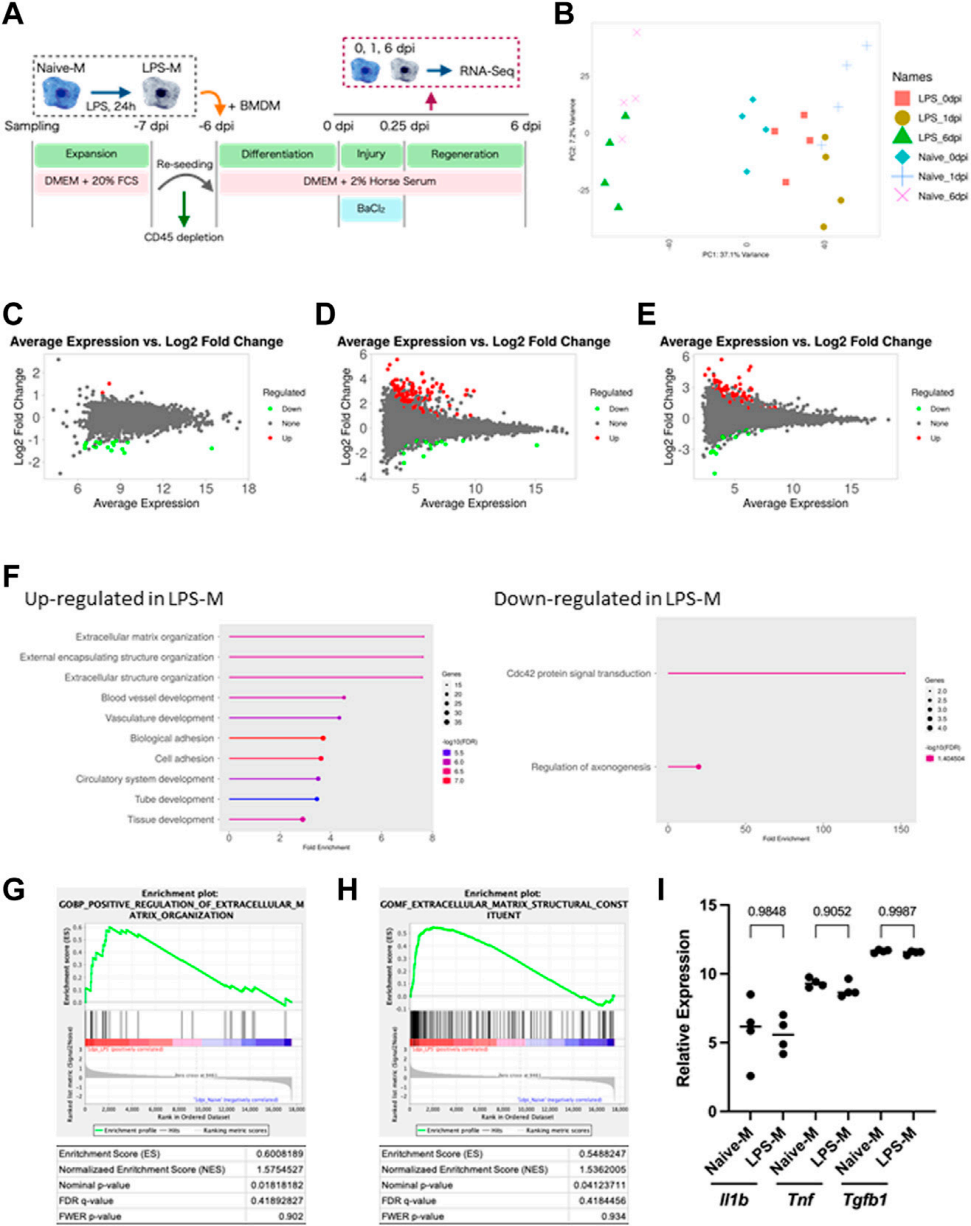
Transcriptome analysis of naive and LPS-pre-stimulated macrophages. **(A)** Schematic representation of the time course and culture conditions. **(B)** Principal component analysis of global gene expression in Naive-M and LPS-M at each time-point. **(C–E)**: MA-plots showing global gene expression of Naive-M and LPS-M at **(C)** 0 **(D)** 1, and **(E)** 6 dpi. DEGs are highlighted in red and green. **(F)** Gene ontology analysis of genes upregulated in LPS-M compared with Naive-M at 1 dpi. Top 20 enriched biological processes are shown. **(G, H)** GSEA of Naive-M and LPS-M at 1 dpi displaying enrichment plot of “Positive regulation of extracellular matrix organization” **(G)** and “Extracellular matrix structural constituent” **(H)**. **(I)** Relative expression of proinflammatory cytokines and *Tgfb1* at 1 dpi. Data are presented as the mean ± standard deviation of biologically independent samples from different mice. DEGs, differentially expressed genes; DMEM, Dulbecco’s modified Eagle medium; dpi, days post-injury; GSEA, gene set enrichment analysis; Tnf, tumor necrosis factor alpha; Il1b, interleukin 1 beta; Tgfb1, transforming growth factor 1 beta.

We performed transcriptomic analysis to determine the mechanisms involved in the reduced contribution of macrophages to skeletal muscle regeneration due to LPS pre-stimulation. Ly6G^−^CD45^+^CD11b^+^ Macrophages were sorted by flow cytometry at 0, 1, and 6 dpi. Then, gene expression in Naive-M and LPS-M was detected using RNA sequencing (Figure 4A). Principal component analysis of global gene expression in the aforementioned six samples demonstrated a more distinct gene expression pattern ofNaive-M and LPS-M at 1 dpi than at 0 and 6 dpi (Figure 4B). Correspondingly, the number of DEGs identified between Naive-M and LPS-M were highest at 1 dpi for 134 genes compared to 0 dpi for 19 genes and 6 dpi for 108 genes (Figure 4C–E). The relatively more dynamic changes in gene expression patterns at 1 dpi indicated that LPS stimulation was associated with a significant difference in macrophage responsiveness to skeletal muscle injury. We performed gene ontology analysis on both sets of DEGs to assess the biological process responsible for the difference in skeletal muscle regeneration efficiency based on the hypothesis that the difference in reactivity immediately after skeletal muscle injury causes the difference. The genes involved in extracellular matrix (ECM) organization were the most enriched among those upregulated in LPS-M (Figure 4F). Positive regulation of ECM organization and expression of their components were also confirmed by GSEA (Figures 4G, H). The differential expression of these ECM components was considered a muscle injury-specific response, because Naive-M and LPS-M showed no difference in the expression of ECM-associated genes when BaCl_2_ was directly added without co-culturing with skeletal muscle (Supplementary Figures S7A–D). In contrast, there was no decrease in the expression of proinflammatory cytokines generally observed in LPS-stimulation (Seeley and Ghosh, 2017) (Figure 4I). There was also no major difference in the expression of *Tgfb1* encoding transforming growth factor beta (TGF-β), which is released by macrophages during regeneration and indirectly promotes tissue remodeling (Sass et al., 2018), between Naive-M and LPS-M (Figure 4I). These results suggest that LPS-stimulated macrophages produce excessive amounts of ECM substrates immediately after skeletal muscle injury, which decreases their regenerative efficiency. We also showed that the gene expression pattern altered by LPS stimulation immediately after skeletal muscle injury was distinct from that altered by secondary stimulation with endotoxin.

## 4 Discussion

In this study, we successfully established an *in vitro* skeletal muscle regeneration model without the use of specialized equipment and techniques. This model showed macrophage-dependent changes in regeneration efficiency. Using this model, we also showed that LPS pre-stimulation in macrophages reduces the efficiency of skeletal muscle regeneration. Indeed, the gene expression pattern of the LPS-stimulated macrophages was found to be significantly different upon exposure to skeletal muscle injury compared to naive macrophages. These results indicate that our 2D model provides a quantitative evaluation system for muscle damage and regeneration. Unlike 3D culture systems and animal models, this model is a simple and high-throughput evaluation system that we believe will be useful for various applications, such as high-throughput compound screening and *in vitro* pathological modeling.

Owing to the importance of the microenvironment and spatial organization, three-dimensional culture systems have been used to simulate skeletal muscle regeneration *in vitro* (Juhas et al., 2014; Juhas et al., 2018; Fleming et al., 2019; Fleming et al., 2020; Tiburcy et al., 2019). Previously reported 2D culture systems have been unable to fully reproduce the results of *in vivo* experiments (Fleming et al., 2019; Davoudi et al., 2022). These culture systems consist of myoblasts alone and their incomplete reproduction suggests the importance of evaluating an appropriate microenvironment for studying skeletal muscles. Based on this, we attempted to construct a microenvironment similar to that *in vivo* by transferring all the cells obtained from the skeletal muscle tissue of mice to an *in vitro* culture system. As a result, we found that the presence or absence of macrophages, one of the cells that construct the microenvironment, affects the efficiency of regeneration, and succeeded in reproducing skeletal muscle regeneration to a comparable level to pre-injury without supplementation of myogenic cells and specific myokine expression that could not be done by conventional 2D model (Fleming et al., 2019; Davoudi et al., 2022). These results emphasize that accurate construction of the microenvironment is crucial for complete reproduction, even in 2D culture systems. However, in this study, we did not identify the types and proportions of cells in the culture environment. Furthermore, even if all cells constituting skeletal muscle tissue were obtained in our model, the microenvironment was not fully reproduced because of selection based on their growth rate and culture environment. Thus, tight regulation of the type and number of cells constituting the skeletal muscle microenvironment will more accurately recapitulate heterogeneous cell-cell interactions and uncover novel mechanisms that might be masked by the limitations of this model.

In this model, macrophage-dependent increase in regenerative efficiency was observed. One possible reason for this is phagocytosis of debris by macrophages. During tissue injury, macrophages contribute to more efficient tissue regeneration by phagocytosis for clearance of the injured site (Wynn and Vannella, 2016). In our model, the number of macrophages in the culture environment correlated inversely with the area of debris (Figures 2E, H) suggesting that phagocytosis of debris may have contributed to the efficient regeneration. Another possible reason is the increased inflammatory response due to the presence of macrophages in culture environment (Figures 1E, F). The inflammatory response induced during skeletal muscle injury is known to promote myoblast proliferation, which is important role for more efficient regeneration (Yahiaoui et al., 2008; Hoene et al., 2013). In previous reports, the limitation of skeletal muscle regeneration models using myoblasts alone was thought to be due to a limited inflammatory response (Fleming et al., 2019), and the macrophage-incorporated type showed an increased inflammatory response in whole culture environment (Juhas et al., 2018). Therefore, the presence of macrophages in this model may have augmented the inflammatory response during skeletal muscle injury and contributed to myoblast activation. Further analysis is needed to determine if this supposed phenomenon contributed to more efficient skeletal muscle regeneration in this model.

In our data, macrophages stimulated with LPS exhibited decreased efficiency in skeletal muscle regeneration and showed impaired elimination of injury site debris. Interestingly, several previous studies have suggested a correlation between fibrosis caused by ECM overaccumulation and decreased phagocytosis of infiltrating macrophages, which occurred simultaneously (Zhang et al., 2019; Al-Zaeed et al., 2021). However, the detailed molecular mechanisms supporting this correlation are not clear. Our results showed that pre-stimulation with LPS inhibited debris elimination, but the transcriptome results did not reveal significant differences in the phagocytic pathway of the macrophages themselves. One possibility supporting this result is the reduced migratory capacity of macrophages due to excessive accumulation of ECM. Since macrophages infiltrating tissue are known to change their migration speed depending on the density of the ECM (Gao et al., 2021), it is suggested that pre-stimulation of LPS may have limited macrophage access to debris through the excessive accumulation of ECM.

Innate immune memory influences subsequent immune responses via epigenetic changes in innate immune cells caused by primary stimuli such as cytokines and exogenous antigens (Domínguez-Andrés and Netea, 2020). LPS is one of the representative ligands that induce innate immune memory, and it is known that repeated stimulation of LPS cannot reproduce the gene expression that occurs with the first stimulation due to the epigenetic changes it causes (Foster et al., 2007). In addition, damage-associated molecular patterns (DAMPs) released upon tissue injury function as ligands for Toll-like receptor 4 (Gong et al., 2020) and they can also form an innate immune memory (Jentho and Weis, 2021; Bhattarai et al., 2022). Therefore, it is suggested that the altered gene expression between LPS-M and Naive-M observed in this study are associated with epigenetic changes in macrophages caused by prior LPS stimulation that altered the response to DAMPs released from injured skeletal muscle. Based on our findings and several reports, we suggest the possibility that Toll-like receptor 4-mediated innate immune memory induces abnormal ECM production by macrophages, which prevents complete tissue regeneration. Consistent with our transcriptome results, demethylation of genes involved in ECM formation was most enriched in epigenetic changes due to innate immune memory formed by damage-associated molecular patterns released by the Duchenne muscular dystrophy model mice (Bhattarai et al., 2022). Furthermore, the mechanism of macrophages involved in fibrosis after tissue injury has been considered indirect and mediated via activating factors such as TGF-β released by them (Sass et al., 2018). However, it has been reported that ECM released by macrophages is directly involved in fibrosis during the regenerative process after heart attack (Simões et al., 2020). Thus, our results suggest that in many diseases with repeated tissue damage, abnormal ECM production by macrophages with innate immune memory is directly involved in fibrosis.

Immediately after infiltration in response to tissue injury, macrophages promote myoblast proliferation by releasing inflammatory cytokines, and as regeneration proceeds, they release anti-inflammatory cytokines that remit inflammation (Chazaud, 2020). Thus, the process of tissue regeneration depends largely on the appropriate timing and amount of cytokine production by macrophages. Because innate immune memory significantly alters the potential of cytokines released by macrophages through its epigenetic changes (Saeed et al., 2014), macrophages taken innate immune memory may have abnormal cytokine production potential during tissue regeneration, disrupting the proper regenerative process. Sarcopenia is an age-related loss of muscle mass, and the mechanism involves a decreased regeneration capacity in skeletal muscle associated with macrophage abnormality (Ferrara et al., 2022). In addition, aging macrophages exhibit significant epigenetic alterations in inflammation-related genes (Dekkers et al., 2019). Therefore, induction of appropriate innate immune memory targeting macrophages and reprogramming their epigenetics may be a promising therapeutic target for sarcopenia. However, the limitation of our study is that the innate immune memory induced by LPS is due to an artificial system and may differ from the actual pathophysiology of the disease, leaving room for elucidation.

## Data Availability

The datasets presented in the study are deposited in the https://www.ncbi.nlm.nih.gov/ repository, accession number PRJNA880816.
