# Supplementary material for: A concise in vitro model for evaluating interactions between macrophage and skeletal muscle cells during muscle regeneration
Source: Front Cell Dev Biol. 2023 May 18;11:1022081. doi: 10.3389/fcell.2023.1022081 (PMC10236217; doi:10.3389/fcell.2023.1022081)
Supplement: Supplementary file 1 [file Table1.DOCX]

Supplementary Material

An *in vitro* model for evaluating interactions between macrophage and skeletal muscle cells during muscle regeneration

**Naoya Kase^1^, Yohko Kitagawa^1^, Akihiro Ikenaka^1^, Akira Niwa^1^and Megumu K. Saito^1**^ Correspondence:** Megumu K. Saito: [msaito@cira.kyoto-u.ac.jp](mailto:msaito@cira.kyoto-u.ac.jp)


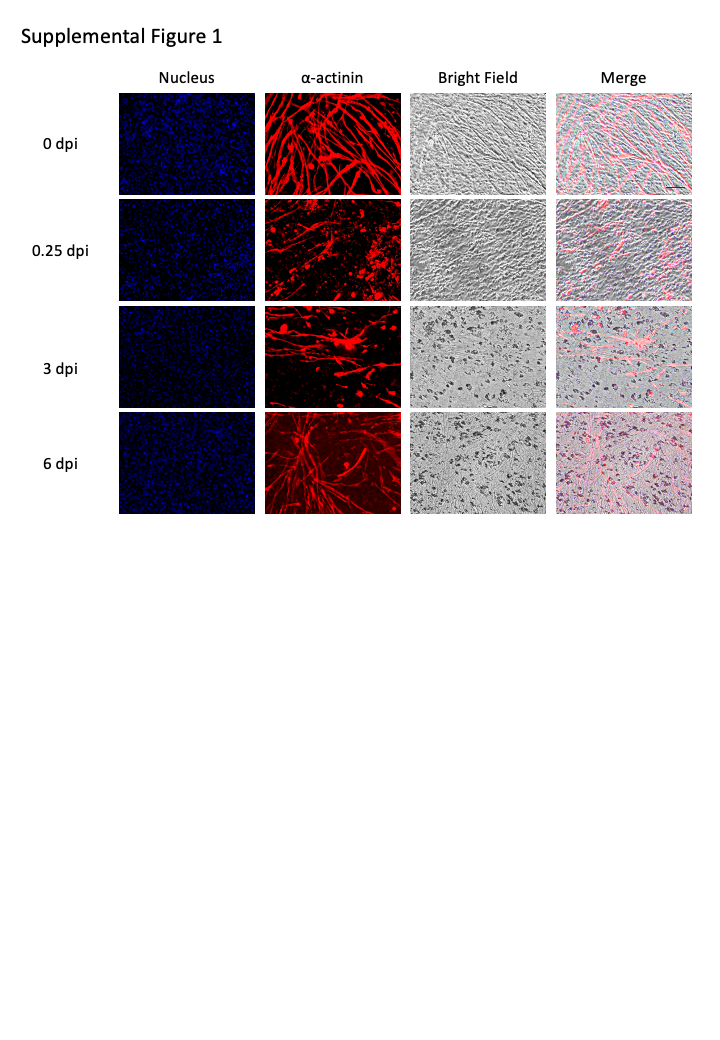


**Supplementary Figure 1.** Transmitted light images and immunofluorescence images of myotube formation at 0, 0.25, 3 and 6 dpi, related to Figure 1A. Scale bar indicates 100 μm.


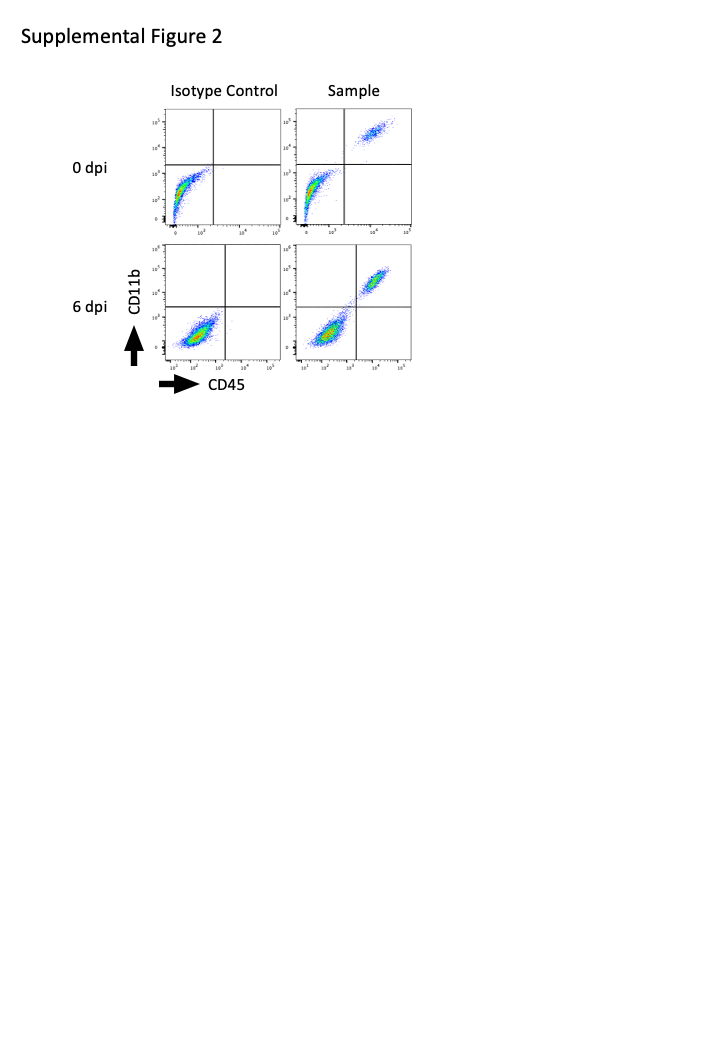


**Supplementary Figure 2.** Flow cytograms for the detection of macrophages at 0 and 6 dpi, related to Figure 2A. To show that the gate strategy is appropriate, plots with isotype control staining were shown. Sample plots are same as in Figure 2A.

**
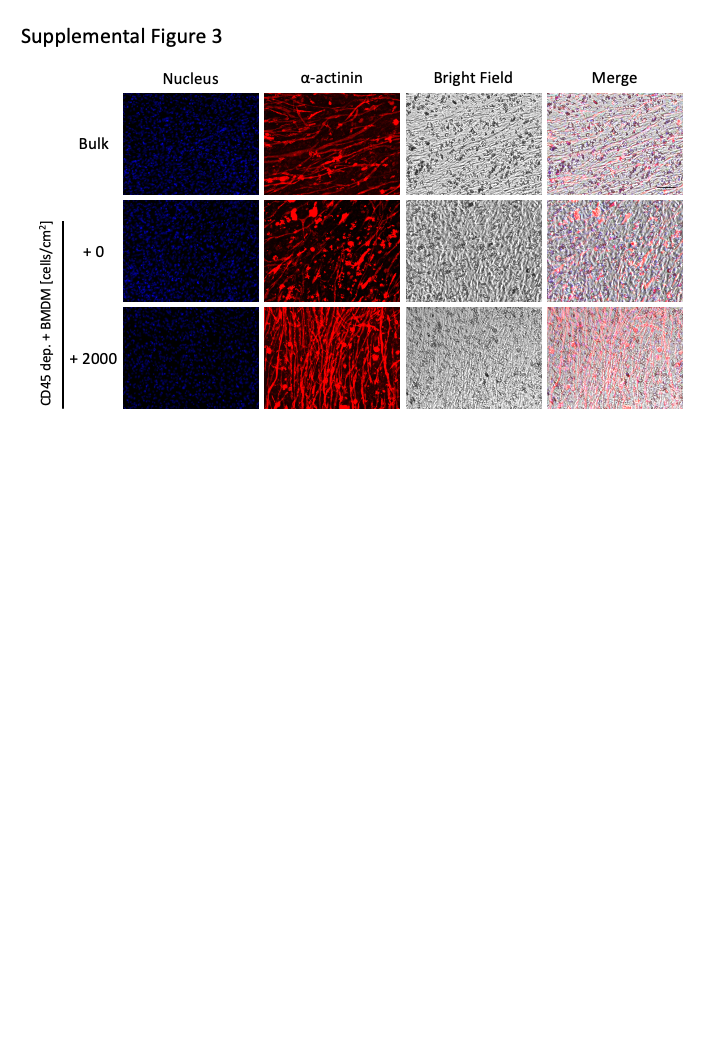
**

**Supplementary Figure 3.** Transmitted light images and immunofluorescence images of bulk condition and macrophage depletion with or without BMDM at 6 dpi, related to Figure 2F. Scale bar indicates 100 μm.

.

**
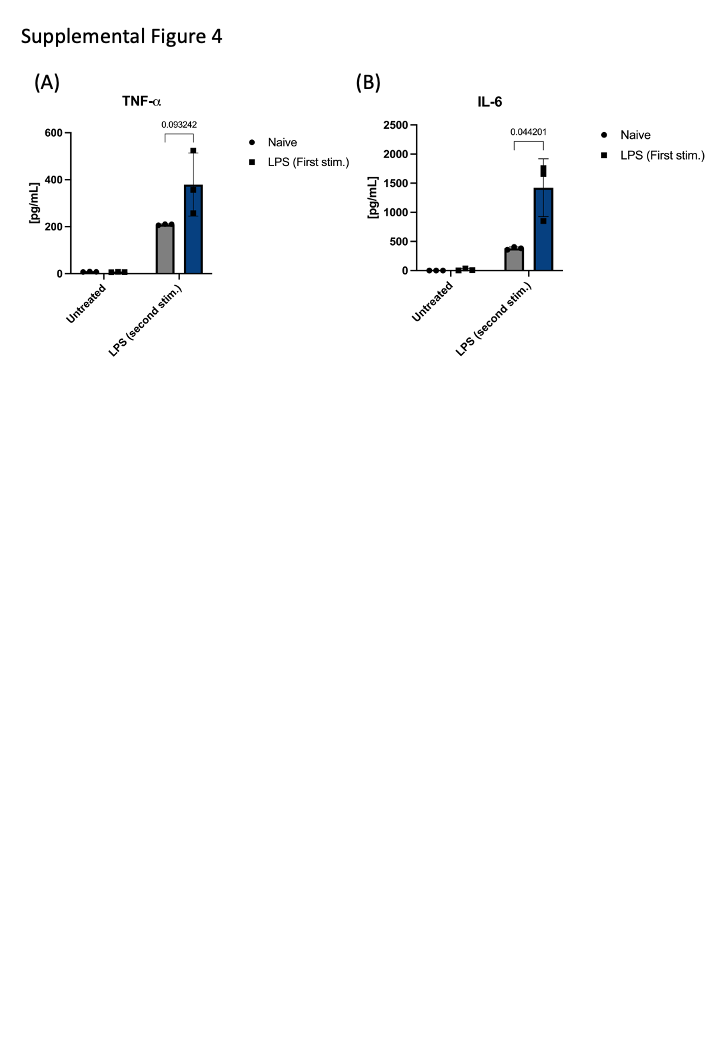
**

**Supplementary Figure 4.** Comparison of LPS-induced secretion of proinflammatory cytokines between unstimulated and LPS pre-stimulated BMDMs. A and B: Quantification of produced (A) TNF-α or (B) IL-6 from BMDMs by second stimulation of LPS detected by ELISA. TNF-α, tumor necrosis factor alpha; IL-6, interleukin 6. Statistical analysis was performed using the Student's t-test. Data are presented as the mean ± standard deviation and P values of biologically independent samples from different mice.


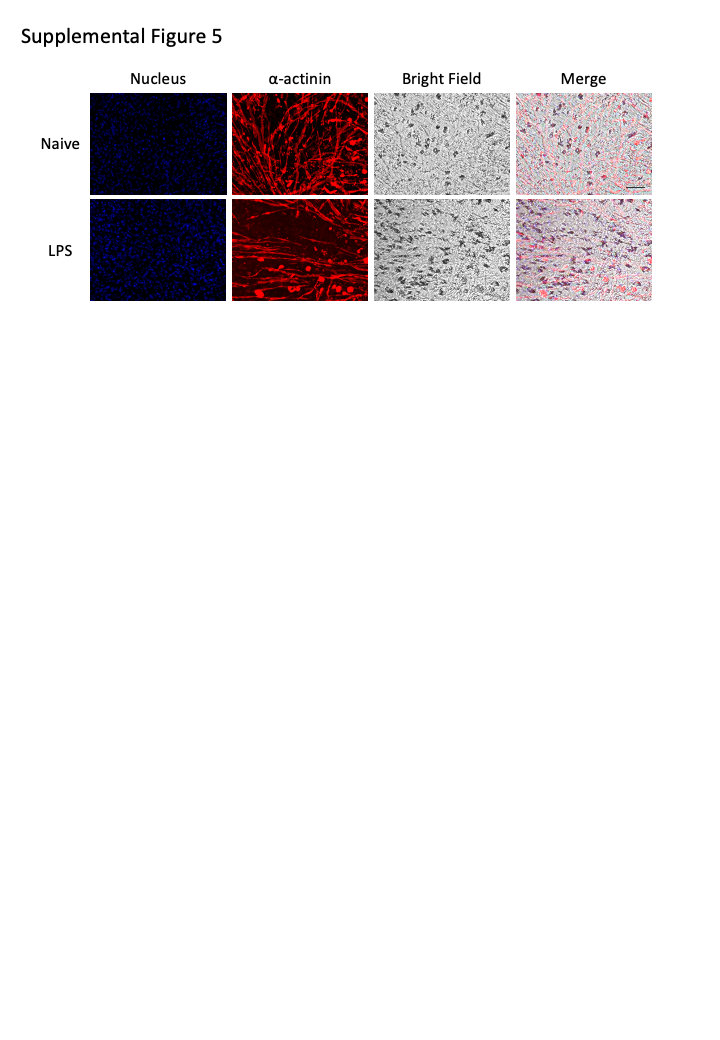


**Supplementary Figure 5.** Transmitted light images and immunofluorescence images of Naive-M and LPS-M condition, related to Figure 3D. Scale bar indicates 100 μm.


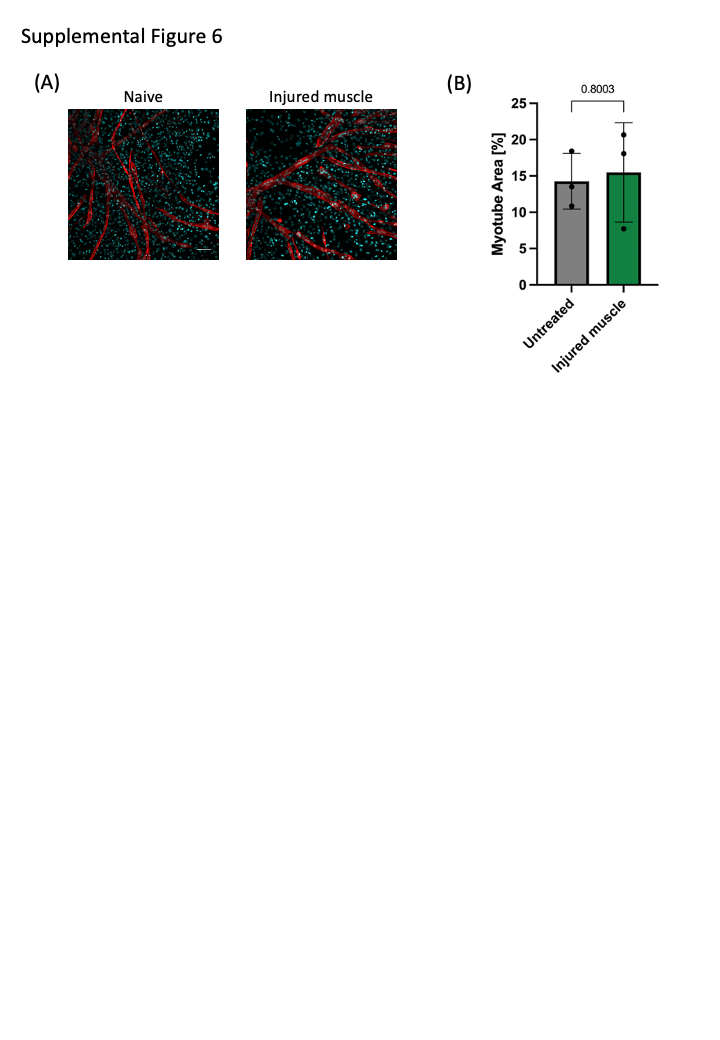


**Supplementary Figure 6.** Evaluation of the efficiency of skeletal muscle regeneration by exposure to factors derived from injured skeletal muscle. A: Representative images of myotube formation after addition of naïve or injured muscle-exposed macrophages at 6 dpi. Scale bar indicates 100 μm. B: Quantification of myotube area (N = 3). Statistical analysis was performed using the Student's t-test. Data are presented as the mean ± standard deviation and P values of biologically independent samples from different mice.


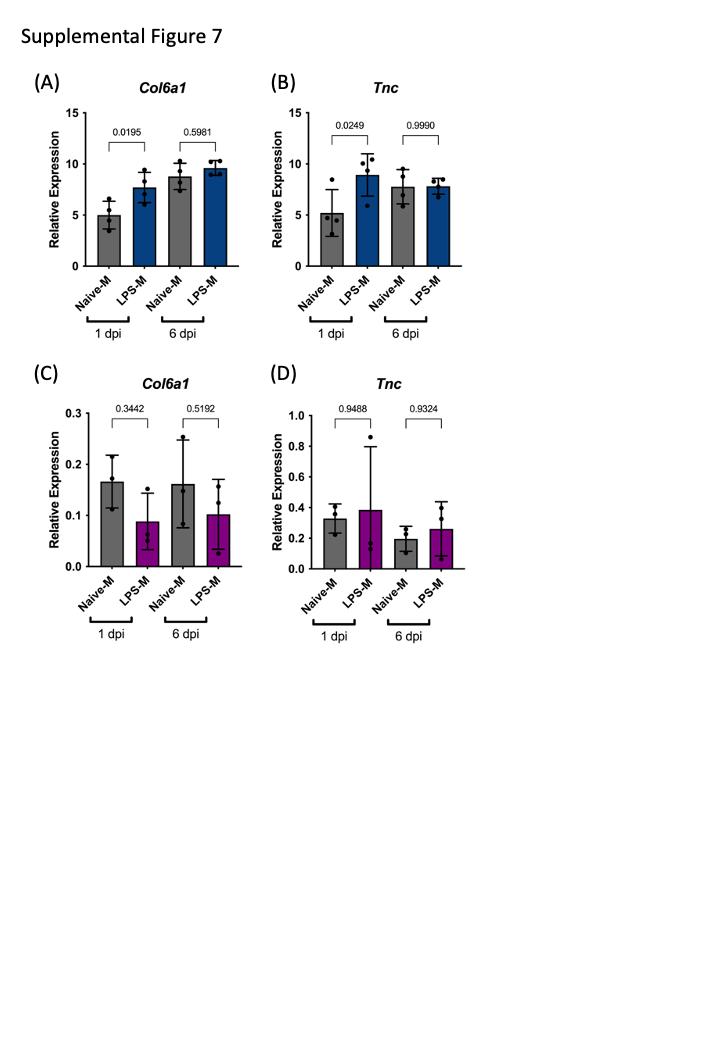


**Supplementary Figure 7.** Gene expressions of ECM components after BaCl2 treatment with or without co-culture to skeletal muscle. A and B: Relative expression levels of (A) Col6a1 and (B) Tnc (N=4) with co-culture to skeletal muscle detected by RNA-seq. C and D: Relative expression levels of (C) Col6a1 and (D) Tnc (N=3) without co-culture to skeletal muscle detected by qPCR. Statistical analysis was performed using one-way ANOVA followed by Šidák post-hoc tests. Data are presented as the mean ± standard deviation and P values of biologically independent samples from different mice. Col6a1, collagen type VI alpha 1 chain; Tnc, tenascin C.
